# Scaling Up Towards International Targets for AIDS, Tuberculosis, and Malaria: Contribution of Global Fund-Supported Programs in 2011–2015

**DOI:** 10.1371/journal.pone.0017166

**Published:** 2011-02-23

**Authors:** Itamar Katz, Ryuichi Komatsu, Daniel Low-Beer, Rifat Atun

**Affiliations:** 1 The Global Fund to Fight AIDS, Tuberculosis and Malaria, Geneva, Switzerland; 2 Business School, Imperial College London, London, United Kingdom; Tulane University, United States of America

## Abstract

**Objective:**

The paper projects the contribution to 2011–2015 international targets of three major pandemics by programs in 140 countries funded by the Global Fund to Fight AIDS, Tuberculosis and Malaria, the largest external financier of tuberculosis and malaria programs and a major external funder of HIV programs in low and middle income countries.

**Design:**

Estimates, using past trends, for the period 2011–2015 of the number of persons receiving antiretroviral (ARV) treatment, tuberculosis case detection using the internationally approved DOTS strategy, and insecticide-treated nets (ITNs) to be delivered by programs in low and middle income countries supported by the Global Fund compared to international targets established by UNAIDS, Stop TB Partnership, Roll Back Malaria Partnership and the World Health Organisation.

**Results:**

Global Fund-supported programs are projected to provide ARV treatment to 5.5–5.8 million people, providing 30%–31% of the 2015 international target. Investments in tuberculosis and malaria control will enable reaching in 2015 60%–63% of the international target for tuberculosis case detection and 30%–35% of the ITN distribution target in sub-Saharan Africa.

**Conclusion:**

Global Fund investments will substantially contribute to the achievement by 2015 of international targets for HIV, TB and malaria. However, additional large scale international and domestic financing is needed if these targets are to be reached by 2015.

## Introduction

In 2000, at the United Nations Millennium Summit the international community committed to achieving the Millennium Development Goals (MDGs) [1] including Goal 4 on reducing child mortality, Goal 5 on improving maternal health and Goal 6 to combat HIV/AIDS, malaria and other communicable diseases such as tuberculosis (TB) with targets for each aimed at addressing the huge burden of disease, which in 2008 led to 2 million (1.7–2.4 million) AIDS deaths [2], 1,318,108 (1,087,802–1,672,447) deaths due to TB [3], and 863,000 (708,000–1,003,000) malaria deaths [4]. These three pandemics account for the majority of morbidity and mortality in low-income countries leading to substantial human suffering and economic loss. In 2005 governments also committed to “Developing and implementing a package for HIV prevention, treatment and care with the aim of coming as close as possible to the goal of universal access to treatment by 2010 for all those who need it” [5].

These commitments have prompted governments, development agencies, new financing institutions such as the Global Fund to Fight AIDS, Tuberculosis and Malaria (the Global Fund), technical organizations, and the civil society to massively scale up investments and life-saving and preventive interventions for addressing these epidemics. These efforts have enabled low- and middle-income countries to expand the number of people receiving antiretroviral (ARV) treatment from 300,000 in 2002 to 5.25 million by the end of 2009 [6]; increase in the number of long lasting insecticidal nets (LLINs) distributed in 35 high-burden African countries from less than 10 million in 2004 to 35–44 million per year between 2006–2008 [4]; and boost the detection rate of new smear-positive tuberculosis cases from 40% (36%–44%) in 2000 to 61% (55%–67%) in 2008 [3].

In spite of these achievements, according to reviews by UN organizations of progress towards the MDGs [7] and other internationally agreed targets [8], the scale-up is not sufficient to meet them (See Box S1 for the targets). However, these reviews did not utilise the latest available information on investment and scale up of service delivery interventions [9]. Hence, an up to date assessment of progress in relation to service delivery targets is timely.

This paper examines the expected contribution to 2011–2015 international targets of three key service delivery indicators, namely, people receiving antiretroviral treatment, new smear-positive TB cases detected and treated under DOTS, and ITNs distributed to people in need by programs in low- and middle-income countries financially supported by the Global Fund.

The Global Fund, an independent international financing institution, was established in 2002 to invest in low- and middle-income countries to rapidly scale up interventions to address AIDS, tuberculosis and malaria epidemics.

In 2008, its share in external global financing amounted to 57% for tuberculosis control and 60% for malaria. In 2007, that for HIV was 23% [10]. Between 2003 and 2010, the institution had disbursed US$13 billion to 140 low- and middle- income countries. Of this, around 61% was allocated to HIV/AIDS, 24% to malaria and 15% to TB.

Earlier analyses of achievements of the programmatic targets of country programs supported by the Global Fund indicated limited contribution relative to internationally agreed targets [10], [11]. However, these analyses predate the substantial scale up of these programs to increase coverage. New data enables reassessment of the contribution of the programs supported by the Global Fund to meet international targets.

## Methods

### Programmatic targets of Global Fund-supported programs

The two sources of programmatic targets of Global Fund-supported programs are performance frameworks of approved proposals. Following negotiation between Global Fund and the principal grant recipient, these are translated into grant agreements with principal grant recipients in countries for a period of five years for implementation in two phases (see Box S2 on target setting, [12]): Phase 1, for two years, and Phase 2, typically for three years. Targets from performance frameworks are used to assess the program performance and provision of continued funding. A detailed performance review at the end of Phase 1 of implementation against the Phase 1 targets agreed at grant signing determines whether funding is provided for Phase 2, the scale of financing provided and the targets to be reached in this phase by the country program supported by the Global Fund.

With targets of on-going grants changing prior phase 2 and new round of proposals are continuously increasing the targets of the entire portfolio, for this paper we project future programmatic targets of Global Fund-supported programs based on past performance. We constructed two scenarios to quantify the targets for the period 2011–2015:

1. Scenario A: for 2011–15 we assumed an annual increase in targets similar to the average annual increase from 2007 to 2010. The data for this period consists of actual results achieved (2007–10). After rounding, this scenario projects annual increases of 560,000 persons receiving ARV treatment, 1.425 million smear-positive tuberculosis cases detected and treated, and 36 million insecticide treated nets distributed.
2. Scenario B: While scenario A includes a relatively long period of four years, it consists of investment and scale up patterns that may not be realised. Therefore, in scenario B we averaged the actual increases achieved in 2007–08 or increases in 2009–10, depending on the indicator. In Scenario B, for number of persons receiving ARVs we averaged the annual increase achieved in the period 2009–10, which was lower than that achieved in 2007–8 (as an increasing number of countries begin to assume domestic financing of ARV treatment). For DOTS, we averaged the increase in the number of cases during 2009–10; a figure which was higher than that achieved during 2007–08. For ITNs we took the average annual increase in 2007–08, which is lower than the scale-up achieved in 2009–10. The figures for Scenario B were translated to annual increments of 500,000 ARV treatments, 1.55 million smear-positive tuberculosis cases detected and distribution of 26 million ITNs.

The projections are based on past trends and assume no increase in the scale of funding, in line with the projected financing for the period in question.

The programmatic targets for smear-positive tuberculosis case detection are presented as cumulative figures. The programmatic ITN distribution targets by Global Fund-supported programs are presented as cumulative figures, but when compared to international targets (described below), we assume a fixed life of 4 years for each net [13]. The ARV targets reflect the number of people *currently* receiving ARVs [14].

### Results reported by programs

Past results (2005–10) of Global Fund-supported programs reported by the end of 2010 were compared with international targets. Grantees are required to periodically report to the Global Fund their progress against targets and in delivering health services. Following external review of the data quality of these programmatic results, they are compared with the programmatic targets within the performance framework and used to inform performance-based funding decisions [15]. Programmatic results relate to people reached with goods and services; e.g. the number of people receiving ARVs, the number of new smear-positive TB cases detected under the DOTS strategy, and the number of ITNs distributed [16]. This allows the Global Fund Secretariat to aggregate results on the major indicators (the progress reports submitted by grant recipients are available at http://www.theglobalfund.org). Some programs report national figures rather than that specifically for grants, as the Global Fund, in line with aid effectiveness principles articulated in the 2005 Paris [17], supports country programs, in many countries providing significant proportion of program funding [10]. In particular, for ARV treatment national figures are used for programs operating at national scale, that are performing well, where data quality is high, and where Global Fund financing significantly contribute to the national efforts. ARV figures are verified with the major agencies, such as the President's Emergency Plan for AIDS Relief (PEPFAR) and WHO, to ensure consistency of measurement and to prevent double counting in jointly supported programs [18] that provided ARV treatment to 3.7 million of the 5.25 million people treated with ARVs in low- and middle-income countries by the end of 2009. Around 1.3 million persons received ARV treatment through programs jointly financed by these two agencies [6].

The international targets for ARV treatment, case detection of smear-positive tuberculosis cases and ITN distribution were based respectively on the targets set by UNAIDS, Stop TB Partnerships, the Roll Back Malaria Partnership and WHO. The methodologies used are detailed in Box S1.

## Results

### ARV treatment targets

**Figure 1:**
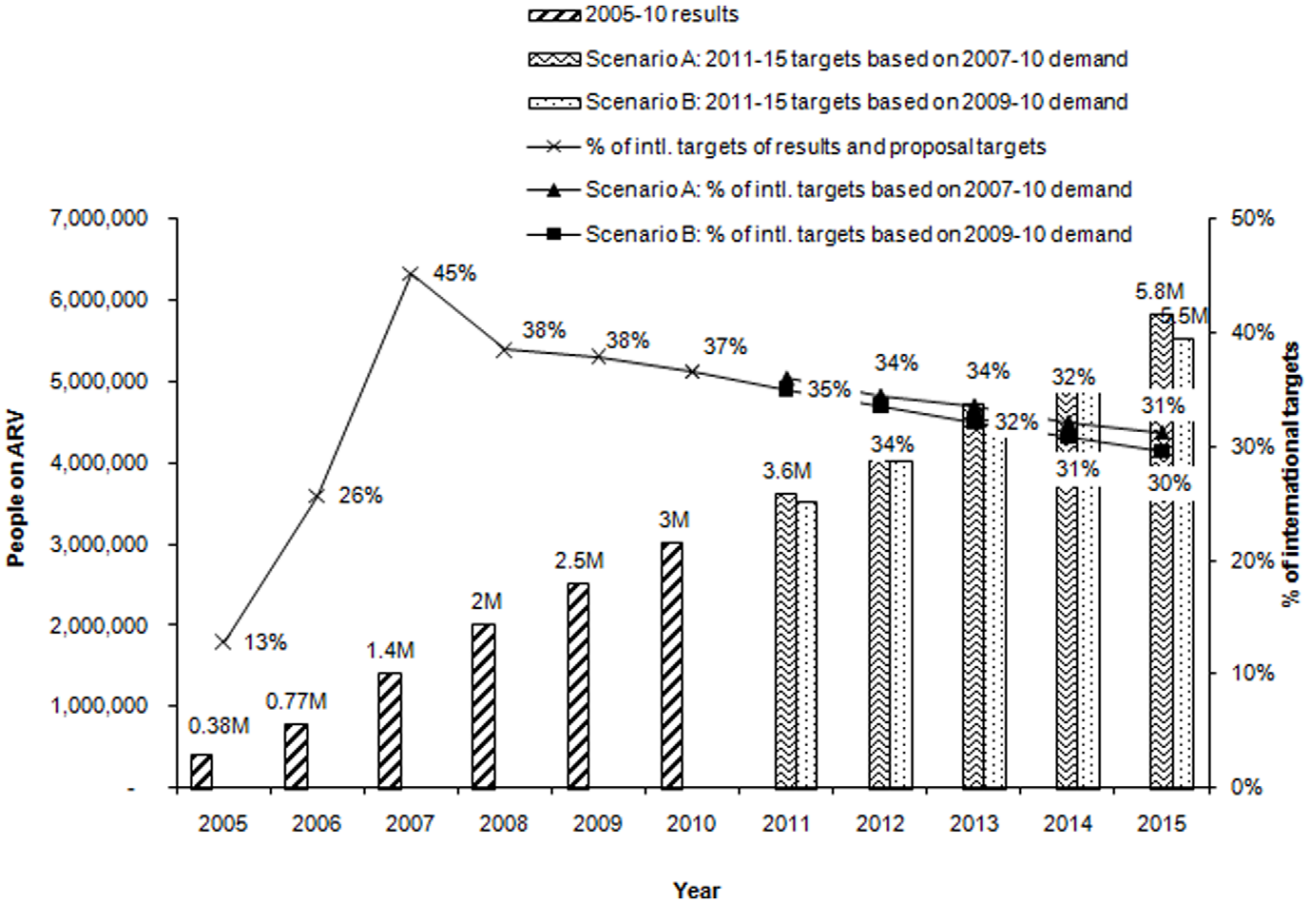
Results and projected targets of ARV treatment by Global Fund-supported programs, 2005 to 2015. Increase in number of people provided with ARV treatment through Global Fund-supported program will be slower than the increase in the need, resulting in a slight decline in their contribution to the international target.

Collectively, the Global Fund-supported programs are projected to almost double the current number of people on ARVs, from 3 million at the end of 2010 to 5.8 million by 2015 if Scenario A (the rate of increase matches that in 2007–10) continues (Figure 1). In Scenario B the pace of increase will be slower, reaching 5.5 million people on ARVs. This is still below the universal access target, which corresponds to 18.6 million people receiving ARVs by 2015. We project that the contribution of Global Fund-supported programs to international ARV targets between 2010 and 2015 will decline from 37% to 31% in Scenario A and to 30% in Scenario B.

### TB case detection targets

**Figure 2:**
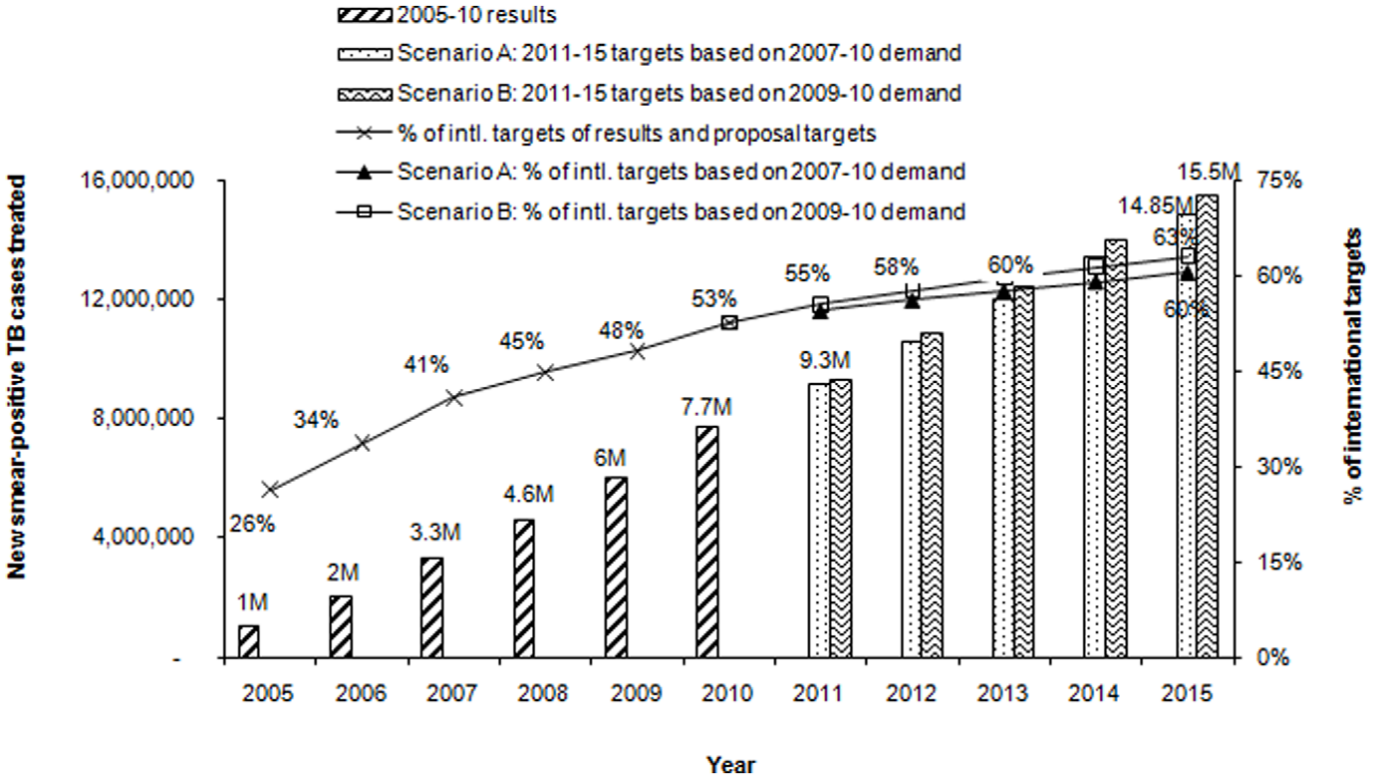
Cumulative results and projected targets of detection and treatment of new smear-positive TB cases by Global Fund-supported programs, 2005 to 2015. With the increase in number of TB cases detected and treated through Global Fund-supported programs, these programs are expected to contribute to 60%–63% of the international target.

Between mid-2004 and end-2010, 7.7 million new smear-positive tuberculosis cases were detected and treated by Global Fund-supported programs. This accounted for 53% of the international target of 14.7 million new sputum smear-positive cases between mid-2004 and end-2010: marking a substantial increase from a contribution of 26% between mid-2004 and end-2005, as shown in Figure 2. Our estimates suggest that cumulatively by 2015 under Scenario A, the Global Fund-supported programs will reach 14.85 million people with new smear-positive TB cases detected and in Scenario B 15.5 million people. As the international target per annum remains largely unchanged from 2007 to 2015 while Global Fund-supported programs increase their annual numbers of new sputum smear-positive TB cases, the contribution of Global Fund-supported targets is expected to increase from 53% in 2010 to 60% in Scenario A and 63% in Scenario B of the international target of 24.6 million new sputum smear-positive TB cases to be treated between mid-2004 and 2015.

### ITN distribution targets

**Figure 3:**
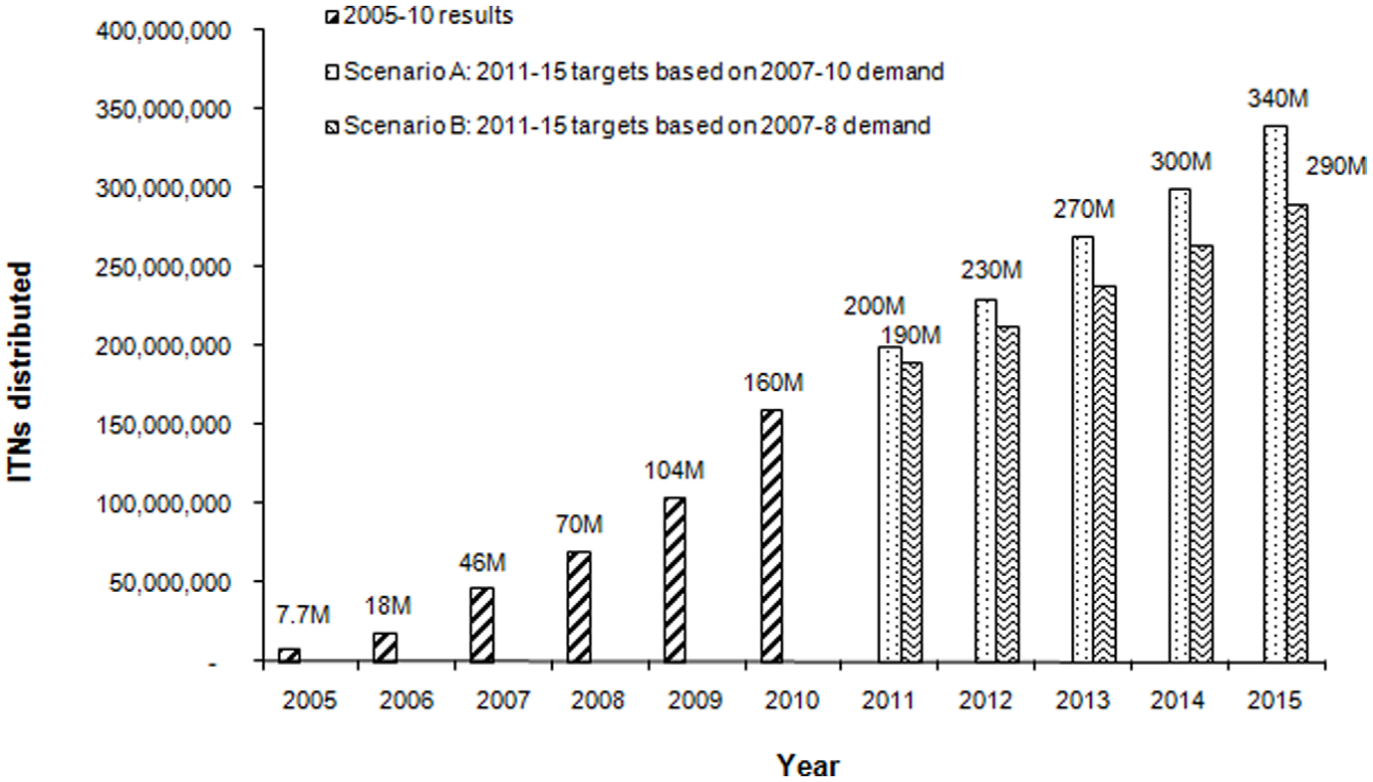
Results and projected targets of ITN distribution by Global Fund-supported programs, 2005 to 2015. Global Fund-supported programs are expected to distribute 290–340 million ITNs between 2003 and 2015.

To prevent malaria, by end-2010, Global Fund-supported programs had distributed 160 million ITNs to people at risk. In Scenario A, 340 million ITNs will be distributed by 2015, while in Scenario B this figure will be 290 million (Figure 3). International targets for ITNs refer to that for sub-Saharan Africa where, as of 2010, around 160 million most-at-risk people (children under-5 and pregnant women) resided in malaria-endemic areas. Achieving Roll Back Malaria Partnership Global Strategic Plan 2005–2015 target of 80% coverage (assuming one ITN needed per person) in 2010 meant distributing 128 million ITNs.

**Figure 4:**
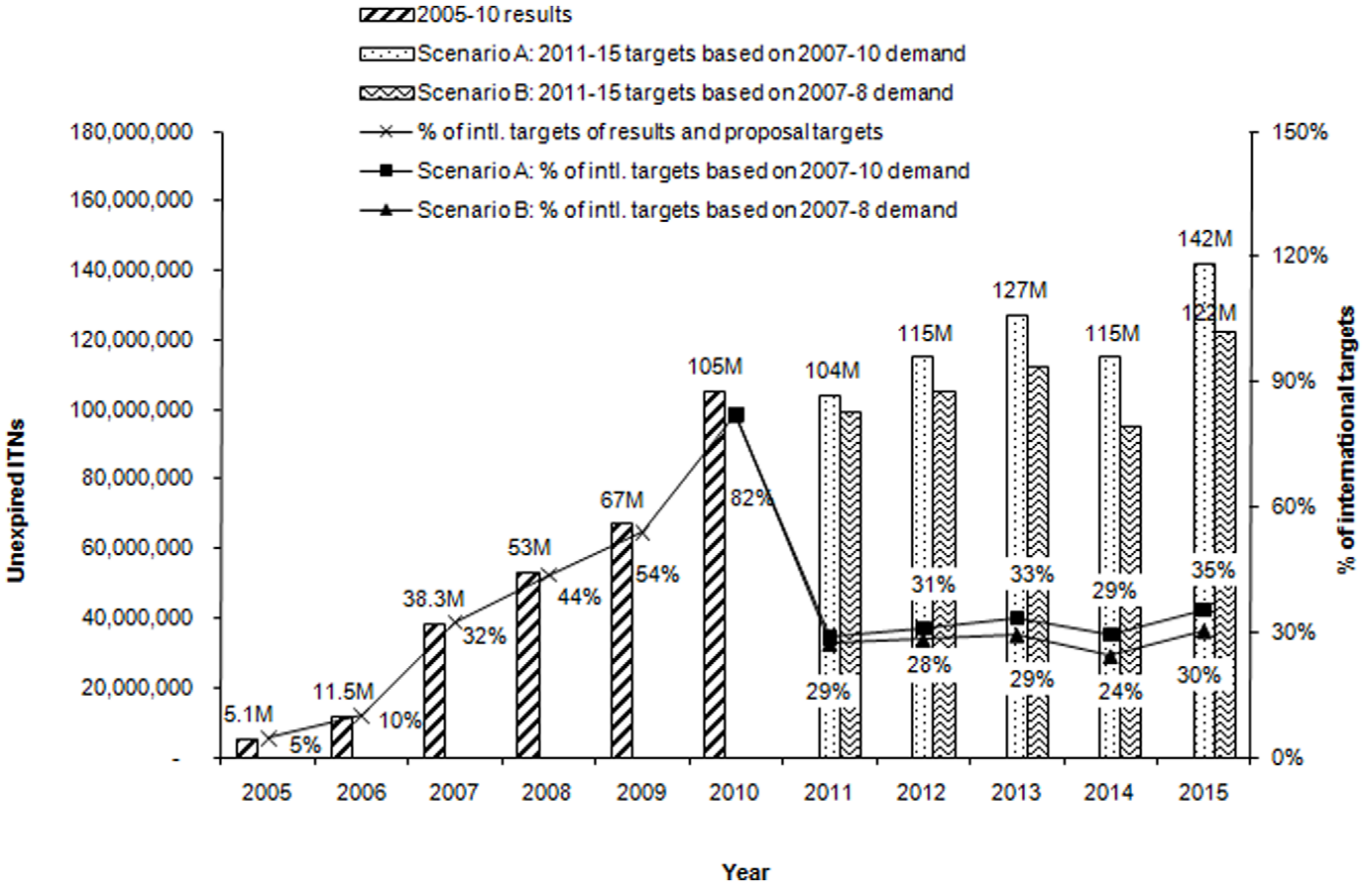
Unexpired ITNs distributed by Global Fund-supported programs in sub-Saharan Africa, 2005 to 2015. In 2010 Global Fund-supported programs have provided over 80% of the international target of ITNs of sub-Saharan Africa. This contribution will reduce to 30%–35% in 2015 with the expansion of the targeted population to all people in risk areas (rather than targeting only pregnant women children under the age of five), and the expiring of ITNs older than 4 years. ITNs to be distributed in 2010 will expire by 2014, and because less ITNs projected to be distributed in 2014 compared to 2010, a decrease is expected between 2013 and 2014.

As detailed in Box S1, from 2011 Global Fund-supported programs will aim to achieve WHO recommendation in 2007 stipulating that all people (not just those at most risk, namely pregnant women and children under the age of five) in risk areas will be covered by ITNs in sub-Saharan Africa, accounting for 402 million ITNs by 2015 [19]. The number of ITNs distributed by Global Fund-supported programs in sub-Saharan Africa increased ten-fold from 5.1 million in 2005 (5% of the international target) to 105 million in 2010 (82%). By 2015, under Scenario A, the number of ITNs delivered in sub-Saharan Africa will increase to 246 million and under Scenario B to 221 million. By 2015, 142 million and 122 million ITNs will still be usable (as they are within their 4-year useful life) under scenarios A and B, contributing respectively to 30% and 35% to the targets for sub-Saharan Africa (Figure 4).

## Discussion

Global Fund-supported programs have rapidly scaled up HIV, TB and malaria services enabling an increase in the number of persons receiving ARVs to increase from 384,000 in 2005, representing 29% of the global results of 1.3 million people on ARVs, to 3 million persons by the end of 2010, representing 37% of the 2010 international targets [11]. However, the increase in targets for reaching universal access targets by 2015 means the Global Fund contribution will decline in Scenario B to 30% and in Scenario A to 31%, requiring substantial increase in domestic and external financing if the targets are to be reached [20] especially as the number of people receiving 2^nd^ line ARVs will increase with increased longevity.

Reaching universal access targets will require a substantial scale-up of comprehensive responses that include prevention, testing, treatment and care [21], [22], even though in some settings the HIV epidemic appears to be stabilizing [2], [23], [24], [25]. Although more correct data has meant a downward revision of HIV estimates [26], addressing the HIV epidemic will require more resources than currently available from international and domestic sources. The resource needs estimated by UNAIDS amount to US$28.4 billion (range US$21.5–34.6 billion) in 2010 and US$49.5 billion (range US$40.9–58.1 billion) in 2015, while the cost of reaching universal access would require US$42.2 billion (US$31.9–51.4 billion) in 2010 and US$54 billion (US$44.6–63.3 billion) in 2015 [27].

The financing of TB control programs in low- and middle-income countries from the Global Fund has substantially increased in recent years, globally the TB prevalence and death rates declining on track to achieve the 2015 targets with the exception of African countries with a high prevalence of HIV [3].

The full implementation of the Global Plan to Stop TB in sub-Saharan Africa and the 22 high-burden TB countries between 2006 and 2015 would result in approximately 14 million lives saved from TB [28]. In sub-Saharan Africa, implementation of the Plan would translate to US$88.1 billion in economic benefits over the same time (compared to no increase in the smear-positive case detection rate from that in 2005). This benefit is almost 9 times higher than the cost of scale-up estimated by the World Bank to be US$10 billion [29].

Global Fund-supported programs were able to distribute 142 million ITNs between 2007 and 2010, compared to 18 million in the period 2002–06. In spite of the scale-up to date and the proposed expansion of Global Fund-supported malaria programs the WHO recommendation for providing ITN coverage to the entire population at risk are not reachable with the current financing levels. Scaling up ITN distribution must be accompanied by behavioral change communications to ensure appropriate and regular use, as owning a bed-net does not readily translate to correct use [30]. On average, correct ITN use at community coverage levels of 60% results in a 50% reduction of uncomplicated malaria episodes and a decline of 5.5 deaths per 1000 children per year in malaria-endemic sub-Saharan Africa [31]. Rapid scale up in ITN distribution will need to be accompanied by health systems strengthening to deliver prevention and treatment to remote rural areas [32], [33], [34], [35]. Expanded coverage by ITNs and ACTs can rapidly reduce malaria associated morbidity and mortality, as has been the case in Zanzibar where a dramatic decline in malaria-associated morbidity and mortality has led to a 10-fold reduction in child mortality [36].

There are several limitations to the data used in this study. Countries set targets in relation to their epidemiological reality, past experience and capability to scale up services. Hence, some targets may be overambitious while others too conservative. To date the results achieved have exceeded the targets set in grants. Future demand might differ from the current levels assumed in this study, for example due to strengthening of the capacity of Global Fund-supported programs over time leading to larger proposals with more ambitious targets. Similarly, international targets may be updated as new targets are agreed. The analysis uses UNAIDS projections as of 2007, prior to 2009 WHO and UNAIDS guidelines of initiating ARV treatment at CD4 below 350, an earlier stage than recommended prior 2009. This increased the estimated number of people in need of ARV treatment from 10 million to 15 million. UNAIDS' 2007 projections partially accounts for the 2009 guidelines, as they provide 82% coverage of those identified 3 years before death, which is equivalent to CD4 count below 350 with WHO's clinical stage 3 [37]. Finally, the projection made for 2011 to 2015 are based on data from current programs, hence the assumption of no increase in funding levels. While funding might increase or decrease, our aim is to show the projected output if there is no change in funding.

Notwithstanding these limitations, our analysis provides the most up-to-date and comprehensive assessment of the programmatic output of Global Fund-supported programs against international targets. In doing so, the analysis provides a more detailed picture on the achievements of the global efforts towards international targets on infectious diseases, including the 6^th^ MDG.

In conclusion, by end of 2010 Global Fund-supported programs implemented by countries had contributed substantially to international targets and will continue to do so to 2015. Universal access by 2010 is currently unlikely, and as demonstrated by this study, at current levels of financing and proposed scale up, achieving universal access targets by 2015 will be difficult [38]. To do so will require countries to set more ambitious performance targets for the programs they implement, and for donors to provide commensurate financing to support scaling up of these programs.

## Supporting Information

Box S1 Estimating international targets.
(DOC)

Box S2 Setting targets within proposals for Global Fund grants.
(DOC)

## References

[1] United Nations (2001). Declaration of Commitment on HIV/AIDS..

[2] UNAIDS, World Health Organisation (WHO) (2009). AIDS epidemic update 2009..

[3] World Health Organisation (WHO) (2009). Global tuberculosis control: a short update to the 2009 report Geneva: WHO.

[4] World Health Organisation (WHO) (2009). World malaria report 2009..

[5] United Nations (2005). 2005 World Summit Outcome..

[6] World Health Organization (WHO), UNAIDS, UNICEF (2010). Towards universal access: scaling up priority HIV/AIDS interventions in the health sector..

[7] United Nations (2009). The Millennium Development Goals Report 2009..

[8] UNAIDS (2007). Financial resources required to achieve universal access to HIV prevention, treatment, care and support..

[9] Murray C (2007). Towards good practice for health statistics: lessons from the Millennium Development Goal health indicators.. The Lancet.

[10] Low-Beer D, Atun R, Grubb I, Sempala MJ, Komatsu R (2009). Scaling Up for Impact.. The Global Fund Results Report 2009.

[11] Komatsu R, Low-Beer D, Schwartlander B (2007). Global Fund-supported programmes' contribution to international targets and the Millennium Development Goals: an initial analysis.. Bulletin of the World Health Organization.

[12] Katz I, Abdel-Aziz M, Olszak-Olszewski M, Komatsu R, Low-Beer D (2010). Factors influencing performance of Global Fund-supported tuberculosis grants.. International Journal of Tuberculosis and Lung Disease (IJTLD).

[13] Miller JM, Korenromp EL, Nahlen BL, Steketee RW (2007). Estimating the number of insecticide-treated nets required by African households to reach continent-wide malaria coverage targets.. Jama-Journal of the American Medical Association.

[14] UNAIDS (2007). United Nations General Assembly Special Session on HIV/AIDS.. Monitoring the Declaration of Commitment on HIV/AIDS. Guidelines on construction of core indicators. 2008 Reporting.

[15] Low-Beer D, Afkhami H, Komatsu R, Banati P, Sempala M (2007). Making Performance-Based Funding Work for Health.. PLoS Med.

[16] World Health Organisation (WHO), UNAIDS, The Global Fund to Fight AIDS Tuberculosis & Malaria, USAID, US Department of State, et al. (2006). Monitoring and Evaluation Toolkit: HIV/AIDS, Tuberculosis, and Malaria..

[17] United Nations (2005). Paris Declaration on Aid Effectiveness.. Ownership, Harmonisation, Alignment, Results and Mutual Accountability.

[18] Boerma JT, Stanecki KA, Newell M-L, Luo C, Beusenberg M (2006). Monitoring the scale-up of antiretroviral therapy programmes: methods to estimate coverage.. Bulletin of the World Health Organization.

[19] World Health Organisation (WHO) (2009). World malaria report 2009..

[20] The African Summit on Roll Back Malaria (25 April 2000). The Abuja Declaration on Roll Back Malaria in Africa, by the African Heads of State and Government..

[21] Low-Beer D, Stoneburner RL (2004). AIDS communications through social networks: catalyst for behaviour changes in Uganda.. African Journal of AIDS Research.

[22] Global HIV prevention working group (2007). Bringing HIV prevention to scale: an urgent global priority.

[23] Shelton JD, Halperin DT, Wilson D (2006). Has global HIV incidence peaked?. Lancet.

[24] Katz I, Low-Beer D (2008). Why Has HIV Stabilized in South Africa, Yet Not Declined Further? Age and Sexual Behavior Patterns Among Youth.. Journal of Sexual Transmitted Diseases.

[25] Asamoah-Odei E, Calleja JMG, Boerma JT (2004). HIV prevalence and trends in sub-Saharan Africa: no decline and large subregional differences.. Lancet.

[26] UNAIDS World Health Organisation (WHO) (2007). AIDS epidemic update: December 2007..

[27] UNAIDS (2007). Financial Resources Required to Achieve Universal Access to HIV Prevention, Treatment, Care and Support..

[28] United Nations, World Health Organisation (WHO) (2006). Global Plan to Stop TB 2006–2015..

[29] Laxminarayan R, Klein E, Dye C, Floyd K, Darley S (2007). Economic Benefit of Tuberculosis Control, World Bank Policy Research Working Paper No 4295..

[30] Korenromp EL, Miller J, Cibulskis RE, Cham MK, Alnwick D (2003). Monitoring mosquito net coverage for malaria control in Africa: possession vs.. use by children under 5 years Tropical Medicine & International Health.

[31] Lengeler C (2004). Insecticide-treated bed nets and curtains for preventing malaria..

[32] Nahlen BL, Low-Beer D (2007). Building to Collective Impact: The Global Fund Support for Measuring Reduction in the Burden of Malaria.. American Journal of Tropical Medicine and Hygiene.

[33] Feachem RGA, Sabot OJ (2007). Global Malaria Control in the 21st Century.. A Historic but Fleeting Opportunity JAMA-Journal of the American Medical Association.

[34] Enserink M (2007). Malaria treatment: ACT two.. Science.

[35] Roberts L, Enserink M (2007). Did the really say.... Eradication?. Science.

[36] Bhattarai A, Ali AS, Kachur SP, Martensson A, Abbas AK (2007). Impact of artemisinin-based combination therapy and insecticide-treated nets on malaria burden in Zanzibar.. PLoS Med.

[37] The eART-linc collaboration writing group (2008). Duration from seroconversion to eligibility for antiretroviral therapy and from ART eligibility to death in adult HIV-infected patients from low and middle-income countries: collaborative analysis of prospective studies.. Sexually Transmitted Infections.

[38] Hayden EC (2008). The AIDS fight: looking ahead to 2010.. Nature.

