# Supplementary material for: Scaling Up Towards International Targets for AIDS, Tuberculosis, and Malaria: Contribution of Global Fund-Supported Programs in 2011–2015
Source: PLoS One. 2011 Feb 23;6(2):e17166. doi: 10.1371/journal.pone.0017166 (PMC3044165; doi:10.1371/journal.pone.0017166)
Supplement: Box S1 — Estimating international targets. (DOC) [file pone.0017166.s001.doc]

**Box S1: Estimating international targets**

***ARV international targets***

The MDGs initially focused on prevention and care, while ARV treatment indicator was added by UNGASS (UNAIDS 2002; United Nations 2009). WHO and UNAIDS have been providing leadership for the “3 by 5” initiative, aiming to provide ARV treatment to 3 million people living with AIDS by 2005. The “3 by 5” initiative resulted in a level of ARV scale-up which was not considered possible before the initiative started in 2003, but still reached less than half of the target by end-2005. From 2005 WHO and UNAIDS are working on universal access for HIV-related services, including ARV treatment.

For future international targets on ARV to 2015, we used the “Phased Scale-Up to Universal Access Scenario” (the phased scenario), one of two scenarios for projecting the number of people on ARV as part of the 2007 UNAIDS resource estimates report (UNAIDS 2007). Aiming to achieve universal access by 2015, this scenario is based on service coverage and capacity by 2006, and consistent with the rate of increase in the last six months of 2006. The phased scenario aims to reach 18.6 million people on ARV by 2015, covering 82% of ARV needs. ARV need is defined as people living with HIV three years before they would die without treatment.

***DOTS international targets***

Halving the prevalence of TB by 2015 is considered to be part of MDG indicators. Essential interim targets to achieve this, and further to virtually eliminate TB by 2050, are to detect 70% of new sputum smear-positive cases and to treat 85% successfully under DOTS programs, particularly for high burden countries by 2005 (Dye, Watt et al. 2005; Dye, Maher et al. 2006). The Stop TB Partnership provides approximate targets for the next decades in line with these MDGs (Stop TB Partnership and World Health Organisation (WHO) 2006), including the estimated targeted numbers of new smear-positive TB patients to be treated to 2015, which were compared with programmatic targets of Global Fund-supported programs. We assumed that availability of financial resources through the Global Fund enables detected new smear-positive TB cases to be treated without delay. The estimated international targets were cumulated from mid-2004 in order to be comparable to targets and results for Global Fund-supported programs (Komatsu, Low-Beer et al. 2007).

***ITN international targets***

The malaria MDG target is to halve the disease burden by 2015. This requires 60% of people at high risk of malaria, namely children under five years of age and pregnant women, to use ITNs by 2005 in sub-Sahara Africa, and African leaders have agreed to this target in Abuja (The African Summit on Roll Back Malaria 25 April 2000). The target has been raised to 80% coverage of people at high risk in the Global Strategic Plan 2005-2015 of Roll Back Malaria partnership (The Roll Back Malaria Partnership 2005). Data on ITN need in Africa, the continent with 89% of malaria deaths (World Health Organisation (WHO) 2009), was obtained from a study estimating it from various national representative or large scale surveys (Miller, Korenromp et al. 2007)

In August 2007, WHO has recommended increasing the ITN coverage to the entire population in risk areas, not just the high-risk groups of children and mothers currently targeted in the coverage targets for sub-Saharan Africa. In the 2009 World Malaria Report, the number of people in risk areas was estimated to be 672 million in 2008. The report further assumed that one ITN will be sufficient for two persons, hence a need for 336 million ITNs (World Health Organisation (WHO) 2009). Given that most proposals analyzed in this paper were developed and submitted before this new recommendation was published and the international targets for sub-Saharan Africa, we assumed these recommendation will be relevant from 2011 onwards.

For the purpose of this paper, country-specific information was extrapolated using population projections to adjust it to population growth (United Nations 2009). ITN distribution targets of Global Fund-supported programs are presented both globally and for sub-Saharan African countries. However, due to data availability on ITN need, only the latter was compared with international targets. In this comparison, expired ITNs, defined here as ITNs older than 4 years, were discounted. On the other hand, 100% usage of distributed bed-nets was assumed, as those not using bed-nets require behavioural change communication rather than additional bed-net.

***References***

Dye, C., D. Maher, et al. (2006). "Targets for global tuberculosis control." International Journal of Tuberculosis and Lung Disease **10**(4): 460-462.

Dye, C., C. J. Watt, et al. (2005). "Evolution of tuberculosis control and prospects for reducing tuberculosis incidence, prevalence, and deaths globally." Jama-Journal of the American Medical Association **293**(22): 2767-2775.

Komatsu, R., D. Low-Beer, et al. (2007). "Global Fund-supported programmes' contribution to international targets and the Millennium Development Goals: an initial analysis." Bulletin of the World Health Organization **85**(10): 805-811.

Miller, J. M., E. L. Korenromp, et al. (2007). "Estimating the number of insecticide-treated nets required by African households to reach continent-wide malaria coverage targets." Jama-Journal of the American Medical Association **297**(20): 2241-2250.

Stop TB Partnership and World Health Organisation (WHO) (2006). Global Plan to Stop TB 2006–2015. Geneva

The African Summit on Roll Back Malaria (25 April 2000). The Abuja Declaration on Roll Back Malaria in Africa, by the African Heads of State and Government. Abuja.

The Roll Back Malaria Partnership (2005). Roll Back Malaria Global Strategic Plan 2005-2015.

UNAIDS (2002). United Nations General Assembly Special Session on HIV/AIDS. Monitoring the Declaration of Commitment on HIV/AIDS. Guidelines on construction of core indicators.

UNAIDS (2007). Financial Resources Required to Achieve Universal Access to HIV Prevention, Treatment, Care and Support. Geneva.

United Nations (2009). The Millennium Development Goals Report 2009. New York, United Nations.

World Health Organisation (WHO) (2009). World malaria report 2009. Geneva.

World Health Organisation (WHO) (2009). World malaria report 2009. Geneva, World Health Organisation.
