# Supplementary material for: Scaling Up Towards International Targets for AIDS, Tuberculosis, and Malaria: Contribution of Global Fund-Supported Programs in 2011–2015
Source: PLoS One. 2011 Feb 23;6(2):e17166. doi: 10.1371/journal.pone.0017166 (PMC3044165; doi:10.1371/journal.pone.0017166)
Supplement: Box S2 — Setting targets within proposals for Global Fund grants. (DOC) [file pone.0017166.s002.doc]

### Box S2: Setting targets within proposals for Global Fund grants

In the process of setting programmatic targets within proposals for Global Fund grants, applicants - and partners, who frequently support applicants in their preparation of proposals - take into consideration the coverage levels of health services they aim to scale-up and the extent they estimate a given additional funding will enable expansion of these health services. As such, targets will differ from one setting to another. However, most grants will end up having ambitious, yet reachable targets. On the one hand, funding is limited and therefore applicants would tend to set ambitious targets to increase the chance their proposal will be approved. On the other hand, targets of proposals are the basis for targets of grant agreements, which failing to achieve them might result in a reduction in the funding. This factor will lead applicants to set realistic targets. Additional factor that ensures ambitious, yet reachable targets is the review of approved proposals during grant negotiations by the Global Fund secretariat. Despite these processes some targets might be modest while other overambitious.
